# T-cell virtuosity in ‘‘knowing thyself”

**DOI:** 10.3389/fimmu.2024.1343575

**Published:** 2024-02-13

**Authors:** Oreste Acuto

**Affiliations:** Sir William Dunn School of Pathology, University of Oxford, Oxford, United Kingdom

**Keywords:** T cell, T cell antigen receptor (TCR), antigen recognition, TCR signalling, allosteric activation

## Abstract

Major Histocompatibility Complex (MHC) I and II and the αβ T-cell antigen receptor (TCRαβ) govern fundamental traits of adaptive immunity. They form a membrane-borne ligand-receptor system weighing host proteome integrity to detect contamination by nonself proteins. MHC-I and -II exhibit the “MHC-fold”, which is able to bind a large assortment of short peptides as proxies for self and nonself proteins. The ensuing varying surfaces are mandatory ligands for Ig-like TCRαβ highly mutable binding sites. Conserved molecular signatures guide TCRαβ ligand binding sites to focus on the MHC-fold (MHC-restriction) while leaving many opportunities for its most hypervariable determinants to contact the peptide. This riveting molecular strategy affords many options for binding energy compatible with specific recognition and signalling aimed to eradicated microbial pathogens and cancer cells. While the molecular foundations of αβ T-cell adaptive immunity are largely understood, uncertainty persists on how peptide-MHC binding induces the TCRαβ signals that instruct cell-fate decisions. Solving this mystery is another milestone for understanding αβ T-cells’ self/nonself discrimination. Recent developments revealing the innermost links between TCRαβ structural dynamics and signalling modality should help dissipate this long-sought-after enigma.

## Building T-cell adaptive immunity

“Immunology: The Science of Self/Nonself Discrimination”, a book by Jan Klein published in 1982 (1), condenses what an immune system normally does, quizzing the biochemical make-up of the host for potential alterations by exogenous or endogenous sources, which reduce fitness and prompt actions to eradicate the causing agent. This central tenet is materialised through biomolecular interactions trained on evolutionary timescales to make binary decisions such as abstaining from reacting to (tolerating) the host’s biomolecules or reacting to unfamiliar ones. Immunity is traditionally divided into innate (or inborn) and adaptive (acquired or combinatorial), which in its most sophisticated form, as discussed here, operates only in jawed vertebrates. Innate immunity is a first line of protection discriminating between the molecular differences in microbial and host nucleic acids, carbohydrates, or proteins that are maintained during the evolutionary time scale (2). Adaptive immunity is a powerful fail-safe system generally set in motion by warnings originating from innate immunity responses (2). Its distinctive character is a vast repertoire of clonally-unique (clonotypic) surface receptors, each with a different ligand-binding site, borne only by lymphocytes. Their characteristic immunoglobulin (Ig)-superfamily fold offers opportunities for molecular recognition of organic polymers, preferentially folded proteins, or small fragments thereof, achieving specificity at single-residue resolution. Quadrillions of diverse binding sites can be theoretically generated by DNA recombination mechanisms involving the juxtaposition of diverse genetically-encoded variable (V), diversity (D), and joining (J) segments and random mutations occurring at the splice sites (3). Such an immense diversity is *de novo* generated during most of the host life span in a ligand-independent fashion and beyond actual needs, like a pro-active plan anticipating an uncertain future. Optimisation and safety processes operate thorough positive selection for clonotypic receptors trained over self-biomolecular structures and deemed apt to bind and signal, and discard those that are highly auto-reactive. The resulting receptor stock is competent for facing nonself entities, predominantly pathogenic micro-organisms. The prodigious chemical and physical diversity afforded by proteins is appropriate for such an undertaking as it provides protein-protein interfaces with a breath of combinatorial of enthalpy-entropy solutions for favourable binding free energy (4) and specific recognition. Contrary to the classical “one receptor one (or few) ligand(s)” paradigm, pairing of clonotypic receptors with ligands requires a process of trial and error to hit an affinity range compatible with a signal delivered to the cell. Depending on their strength, signals can drive lymphocyte homeostasis/survival, death or change of functional fate change for coordinating the removal of a micro-organism or oncoprotein-transformed cell. Binding with adequate affinity and occupancy to nonself ligands (antigens) elicits signals for lymphocyte clonal expansion, a key trait of adapting immunity, ensuring that only receptors that specifically recognise an invading nonself (*e.g.*, a microorganism) are selectively amplified. One fraction of the clonally expanded cell pool is not used for immediate needs but stored as a resting, long-term memory of the specific event, another unique feature of adaptive immunity. Memory cells are selectively expanded upon re-exposure to the same antigen providing faster and more effective protection. These biological marks are shared by B-cells and T-cells that together form the complementary arms of adaptive immunity.

The appearance of primordial components of this extraordinary system about 450 million years ago, manifestly offered considerable survival advantage to jawed vertebrates, as witnessed by the rapid expansion of genes governing adaptive immunity by duplication and diversification into composite families (5). Evolutionary geneticists trace the dawn of adaptive immunity to three founding events: the appearance of Major Histocompatibility Complex (MHC) genes encoding classical class-I and -II proteins (5) (for simplicity, hereafter referred to as MHC-I and-II), genes encoding T-cell antigen receptor (TCR) αβ and γδ dimers and Immunoglobulin (Ig) heavy and light chains that form the B-cell antigen receptor (BCR) and ensuing antibodies (Abs), and, coincidentally, of site-specific recombination-activation genes (RAG1/2) (6). TCR and BCR defined two major lineages of vertebrate lymphocytes that act in concert to protect the host by recognising nonself and organise its neutralisation. Excellent and comprehensive reviews on the origin of adaptive immunity can be found in (5–9).

TCR and MHC-I and -II are membrane proteins presumably originated from a rudimentary receptor-ligand pair involved in cell-cell recognition. Despite being encoded on different chromosomes MHC and TCR co-evolved, witnessing the importance of their interaction for jaw vertebrate fitness (5). TCR α and β together form a variable membrane-distal Ig-like binding site with a definitive preference for targeting the so-called “MHC-fold” (5), the membrane-distal domain of MHC-I and -II. The MHC-fold exhibits highly promiscuous binding with 1:1 stoichiometry of diverse short peptides derived from the degradation of proteins of self or nonself origin. The peptide binding site tolerates single and multiple mutations without compromising protein stability, a property exploited to diversify further the already large repertoire of peptides accommodated by each MHC-I or II allomorph, ranking MHC-I and II among the most polymorphic genes in Chordata (10). Another key founding event in adaptive immunity was the appearance in early jawed vertebrates of mandatory companions of TCRαβ, namely four genes whose products form three smaller dimers (εγ, εδ, ζζ, called CD3). CD3 serves to communicate to the cell interior ligand engagement by TCRαβ, via immunoreceptor tyrosine-based activation motifs (ITAMs). The TCRαβεγεδζζ complex forms an octamer made of four non-covalently bound dimers (11), significantly distinct from hundreds of membrane receptors prototypes successfully tested in evolution for intercellular communication, a singularity that continues to question T-cell biologists.

## TCRαβ ligands: staging self-identity and its modifications

In jawed vertebrates, nucleated cells parade on their surface a significant sample of their individual proteome, such as a “QR-code” granting proof of self-identity. Using intact proteins would be physically and biologically unfeasible. A nifty energy-saving alternative is to employ instead protein surrogates naturally available in great abundance and variety in every cell: peptides issued from the physiological degradation of proteins of every cellular compartment or seized from the extra-cellular milieu (12). A providential gift of evolutionary adaptation for this job was the MHC-fold, born by MHC-I and -II proteins (the latter being the presumed MHC-I ancestor (5). The MHC-fold is the membrane-distal domain of MHC-I α1 chain, stabilised by the β_2_-microglobulin (β_2_m) subunit (13, 14). An analogous MHC-fold in MHC-II arises from the complementation of two “half-MHC-folds” of the α1 and α2 subunits forming a very stable dimer (15). The MHC-fold is made of eight β-strands fashioning a relatively rigid platform (the floor) delimited by two anti-parallel α-helices (the walls), called α_1_ and α_2_ for MHC-I (13, 14) and α_1_ and β_1_ for MHC-II (15). This fold forms a narrow and deep groove (or cleft) of dimensions and chemistry suitable for housing many diverse short peptides. MHC I and II possess molecular signatures for being dispatched near or at protein-grinding machines/compartments (proteosome or endosomal vesicles) where a vast cellular peptidome is generated (12). Generic and dedicated peptidases produce candidate peptides of 8-10 and 10-15 amino acid-long for preferential binding to MHC-I (in the endoplasmic reticulum) (16) and MHC-II (in late endosomal or lysosomal compartments) (17), respectively. MHC-I and -II are assisted by bestowed peptide-loading complexes performing trial and error casting to select peptides that bind with medium-high affinity (K_D_ of 100 to 5 nM (12). Only MHC-I and -II molecules filled with tightly bound peptides are granted access to the PM, thus guaranteeing stable surface presentation of a highly diverse immunopeptidome. Host and microbial proteomes are subject to the same rules of degradation and peptide loading, thus creating a considerable assortment of cellular self-proteome with occasional contamination with peptides derived from non-self proteins (12, 18). A unique feature of MHC-I and -II is their ability to bind large numbers of diverse peptides. A single HLA allomorph can bind 2,000 to 10,000 unique peptides (19), a huge promiscuity apparently incongruous with medium-low nM peptide binding affinities. Crystallographic, mutagenesis and thermodynamic studies explain this apparent paradox (14, 15, 20). Short peptides behave as random coils, yet the entropic cost for accommodating them in the MHC groove in an extended conformation is largely compensated by a dense array of hydrogen-bonding with the peptide main-chain. MHC-I groove is closed at both hands by conserved residues that hook the N- and C-termini of 8-10 amino acid-long peptides via hydrogen bonds. The MHC-II groove has open ends and can bind longer peptides, whose termini extend outside the groove. This generic peptide binding mode alone would make specificity vanishingly small, resulting in loose binding of a good peptide portion and an exceedingly large peptide repertoire, ultimately limiting the ability of TCRαβ to accomplish its task. Imposing some degree of specificity, hence a more frugal choice of peptide diversity is crucial. Indeed, the MHC floor features a few pockets with varying selectivity for certain peptide side chains. Some pockets (generally one per molecular type) are deep and narrow, hence selective for side chains of particular character at some peptide positions that contribute substantially to the binding energy. Other pockets, roomy and shallow accommodate diverse side chains at other positions, adding further contribution for peptide affinity and selectivity. Moreover, the side chains of number of residues in the groove can assume diverse tortional angles for optimal interaction with the peptide, while deep pockets burying peptide side chains afford high hydrophobic and hydrogen bonding energy (20). These few pockets of individual physicochemical character achieve a fair compromise between peptide promiscuity, specificity, and affinity. MHC-I (A, B and C) and MHC-II (DP, DQ, and DR) exhibit the entire human immunopeptidome, which is considerably higher than in a single individual, considering that just for HLA-A and B > 12,000 alleles exist (21). Such huge polymorphism concerns mostly MHC floor and wall residues and much less resides at the top of the α-helices. Peptide residues that cannot bond with floor and walls residues, either interact weakly with the rims of the groove or afford high conformational freedom, both targeted by TCRαβ. 10^5^-10^6^ MHC molecules/cell offer a large mosaic of the individual cellular immunopeptidomes, which, considering all tissues, it represents a spatiotemporal steady-state snapshot of virtually every cellular activity in the organism. This self-panorama is perturbed when MHC-I and/or -II are occasionally loaded with microbially-derived (or mutated oncogene-derived) peptides eventually unmasking the presence of microbial (or mutated host) proteins. While nucleated cells exhibit their own immunopeptidome, specialised innate immunity cells, such as Dendritic cells (DC) residing in critical tissue whereabouts (*e.g.*, in lymph nodes (LNs) display also extracellular peptidomes as they constitutively express MHC-II. DCs possess highly specialised peptide-loading systems for efficient presentation of microbial pathogen-derived or mutated onco-protein immunopeptidome released in the extracellular space. In LNs, DCs select αβ-T cells for the ability to distinguish self and nonself immunopeptidomes. The latter are usually very scarcely represented before host acute morbidity manifests, making hard at this stage for αβ-T cells to perceive them among a vast sample of self-peptides and to engage in rapid and potent countermeasures to prevent chronic morbidity or death.

## TCR αβ diagonal binding to p-MHC

When undertaking a cursory glimpse, TCRαβ and Ig binding sites look alike. Both V domains feature similar β-strands sandwich scaffolds with bulging loops forming analogous complementarity-determining regions (CDRs) 1, 2, and 3 making up the ligand binding site, with the CDR3s centrally located. TCRαβ and BCR ligand binding sites can attain a comparable huge magnitude of diversity (> 10^15^) by similar DNA recombination rules assembling analogous V, D, and J gene segments (22). As for BCR, TCRαβ CDR1, and CDR2 are encoded by germline V segments organised into families (in human ≈ 70 Vα divided into 41 families and ≈ 47 Vβ divided into 23 families) and the CDR3s arise from the somatic juxtaposition of V-J (67 Jα) or V-D-J (13 Jβ and 2 Dβ) segments that substantially augment binding site diversity by imperfect joining and template-independent nucleotides additions. However, similarities stop here as MHC-restricted recognition of peptides implies that Vα and/or Vβ should possess structural signatures virtually absent in V_H_V_L_ binding sites. Indeed, genetic manipulation in mice indicates that MHC restriction is encoded by TCRαβ genes (23, 24). Unlike Abs, TCRαβ diversity in CDR3s is much higher than in CDR1 and CDR2, which feature conserved residues involved in MHC binding (25), incidentally making affinity maturation by somatic hypermutation afforded by Abs prohibitive for TCRαβ. Unlike the BCR, TCRTCRαβ does not have a soluble form. The structural principles of p-MHC recognition by TCRαβ have been largely clarified by crystal structures (15, 26, 27) and reviewed in (28–30). Thus, Ab binding sites exhibit considerable shape variability, typified by geomorphic grouping as cave, crater, canyon, valley, and plain (31), and high binding complementarity often achieved by affinity maturation (32, 33). Catalytic Abs that detect chemical reaction transition states are another illuminating example of Ab binding site structural malleability. The TCRαβ binding site is instead relatively flat with mild undulations and slightly protruding CDR3s (28, 29). Wiley and co-workers vividly portrayed TCRαβ positioning over ligand as suspended over the edges of the MHC groove delimited by two high peaks of α-helices, with bulging CDR3s trying to catch peptide side chains arising from the bottom (27). Most notably, TCRαβ is invariably orientated diagonally with respect to the peptide long axis, an emplacement that maximises the chances for the hypervariable CDR3s to contact the peptide, the most variable portion of the ligand surface. CDR3s focus on the peptide centre but at times on more C- or N-terminally positioned residues. The relatively exiguous peptide surface makes Vα and/or Vβ CDR3s often contacting also MHC residues (26, 27, 30), though alone are unlikely to be the major drivers for the diagonal orientation. Rather, Vα and Vβ CDR2s, which are symmetrically distal from the CDR3s, often contact exclusively conserved residues of MHC-I α2 and α1 (or with MHC-II β1 and α1), respectively. CDR1 loops of Vα and Vβ second systematically a hybrid role by contacting peptide eccentric, N- and C-terminally, residues, as well as MHC-I α2 and α1 (or MHC-II β1 and α1), respectively. A few Vα and Vβ framework residues and, so-called CDR4 loops, occasionally contribute to ligand contacts. The angle of diagonal orientation (or crossing angle) varies considerably in different complexes, though hardly exceeding certain limits (27°≤ θ ≤70°) (34), such that Vα invariably sits on the taller and broken α-helix, whereas Vβ prefers the lower, shorter and smoother α-helix. The different elevation of the MHC α-helices over the groove results in a characteristic tilting (or incident) angle between TCRαβ and ligand that can vary in different complexes (0°≤ θ ≤ 25°) (34). Rare complexes showing limited TCRαβ contacts with peptide or unconventional orientations have been reported (26, 30, 35–37). Variation of diagonal and tilting angles, and of register and extent of peptide contacts suggests that, while systematically zeroing in on MHC, TCRαβ binding site exploits many opportunities for subtle or overt adjustments aimed to augment specificity for the peptide and ligand affinity. Binding promiscuity favoured by roomy shape complementarity and electrostatic interactions between pairs of conserved residues in MHC α-helices and Vα and/or Vβ families may promote an initial docking phase that imposes limits to the orientation of TCRαβ over p-MHC. However, such docking leaves scope for additional energy contributions offered to CDR3s by the physicochemical nature of the peptide and by subtle re-adjustments of all CDRs bonding with MHC (30, 38, 39). Diagonal orientation and binding tuning negotiate MHC restriction and peptide specificity compatible with affinities that elicit receptor signalling of biological relevance, the latter being the definitive arbitration of ligand effectiveness (EC_50_). p-MHC-TCRαβ binding geometry may help explain the TCRαβ-CD3 signalling mechanism (discussed below). Consistent with some earlier suggestions on self-recognition, a few structures of TCRαβ in complex with self p-MHC show limited peptide contacts, yet conserve diagonal orientation (26, 35). In agreement with gradual binding adjustments, thermodynamics, and structural data have suggested considerable conformational changes occurring at the p-MHC-TCRαβ binding interface, indicative of an enthalpically-driven reaction with considerable entropic penalty, reflected by agonist weak/medium solution K_D_ (200 - 0.05 µM), as estimated by surface plasmon resonance using monomers of TCRαβ and p-MHC extracellular domains (40–47). However, evidence for both enthalpy and entropy-driven binding have also been documented (48, 49). 2D affinity measurements in intact T cells using artificial membrane-tethered p-MHC show 100 times higher on-rates and no or some off-rates increase (50, 51), resulting in considerable K_D_ reduction, a realistic estimate of ligand potency compounding the membrane tethered nature of ligand-receptor pairs and stochastic noise. However, KD or the combination of KD and t_1/2_ alone does not satisfactorily explain ligand potency in general and especially for high K_on_ and K_off_ (discussed in 43), leading to the proposal of an “aggregate occupancy dwell-time” by fast rebinding of TCRαβ to the same p-MHC (43, 52). Diffusion-influenced reaction, co-receptors, signal decay slower than ligand unbinding (43), but also enhanced membrane tethering by “short” receptor-ligand pairs, as well as the potential for very fast ligand-mediated TCR-CD3 activation and tight clustering of signalling TCR-CD3 (the latter two discussed below) may be invoked to explain how signals emanating from TCR-CD3 lead to T-cell activation.

Building on previous discussion of MHC-restriction, we will now briefly consider the process that moulds TCR αβ clonotypic repertoires restricted to MHC. Only αβ T-cell precursors reacting sufficiently with p-MHC presented in the thymus environment of the host will generate signals sufficient to survive (positive selection) and further pruned of TCRαβ reacting too strongly to tissue-specific host self p-MHC (negative selection). This mechanism forges immunologically competent αβ T cells that exhibit TCRαβ clonotypes with recognition patterns for self p-MHC at low affinity that will compete with each other for cell survival. Teleologically, the thymic learning process allows to “memorise” the molecular thyself of Socratic wisdom a process that inevitably results in awareness of nonself. In LNs αβ T cells are continuously reminded of such precept by self-pMHC that induce weak signals above background (“tonic” signals). Tonic signal is vital for phenotypic stability and survival of αβ T cells (53, 54), incidentally demonstrating that the peripheral T cells pool is inherently self-reactive. 2D binding data of self-reactive TCRs in intact cells show amazingly short dwell times of ≈100 ms or less (55), yet sufficient to elicit signals that positively select thymocytes and αβ-TCR cell survival. This αβ T cell pool should be cross-reactive to the host immunopeptidome, as weak signalling elicited by a single self-molecular species of the huge immunopeptidome repertoire might be insufficient to ensure αβ T cell survival. Diffused TCRαβ cross-reactivity should be essential for responding to a much larger universe of potential nonself peptides (56), using parsimoniously a TCRαβ clonotypic repertoires estimated in humans to be ≅ 10^11^ out of total number of 10^12^ αβ T cells (57). Such vast cross-reactivity arises from the considerable molecular plasticity of p-MHC -TCRαβ binding (58). Various mechanisms are in place to ensure that self-reactivity never exceeds accidentally a threshold that might result in auto-immunity. This is a particularly delicate exercise as negative feedback of TCR-proximal signalling responsible for signal thresholding are closely connected to positive feedback, whose activation allows to surmount signal threshold (59). Coupling of these devices likely constitutes prototypical steps of a proof-reading signalling regime thought to implement ligand discrimination (60) and determine the sigmoidal trend of αβ T-cell responses to ligand dose.

## p-MHC-induced TCR-CD3 activation

### Spatiotemporal organisation

As for many other membrane receptors, TCR-CD3 signal amplitude and duration depend on ligand affinity and concentration, which together provide non-linear signal inputs (59). Signals of varying intensity (or strength) can generate different scales of cellular responses that may result in cell fate changes (61, 62). Weak agonists (*i.e.*, self p-MHC) support survival of αβ-T-cell precursors during thymic development (63, 64) and of peripheral αβ-T cells (53, 54). αβ-T-cell proliferation, differentiation, and exhaustion, or thymocyte negative selection are induced by strong p-MHC agonists.

**Figure 1:**
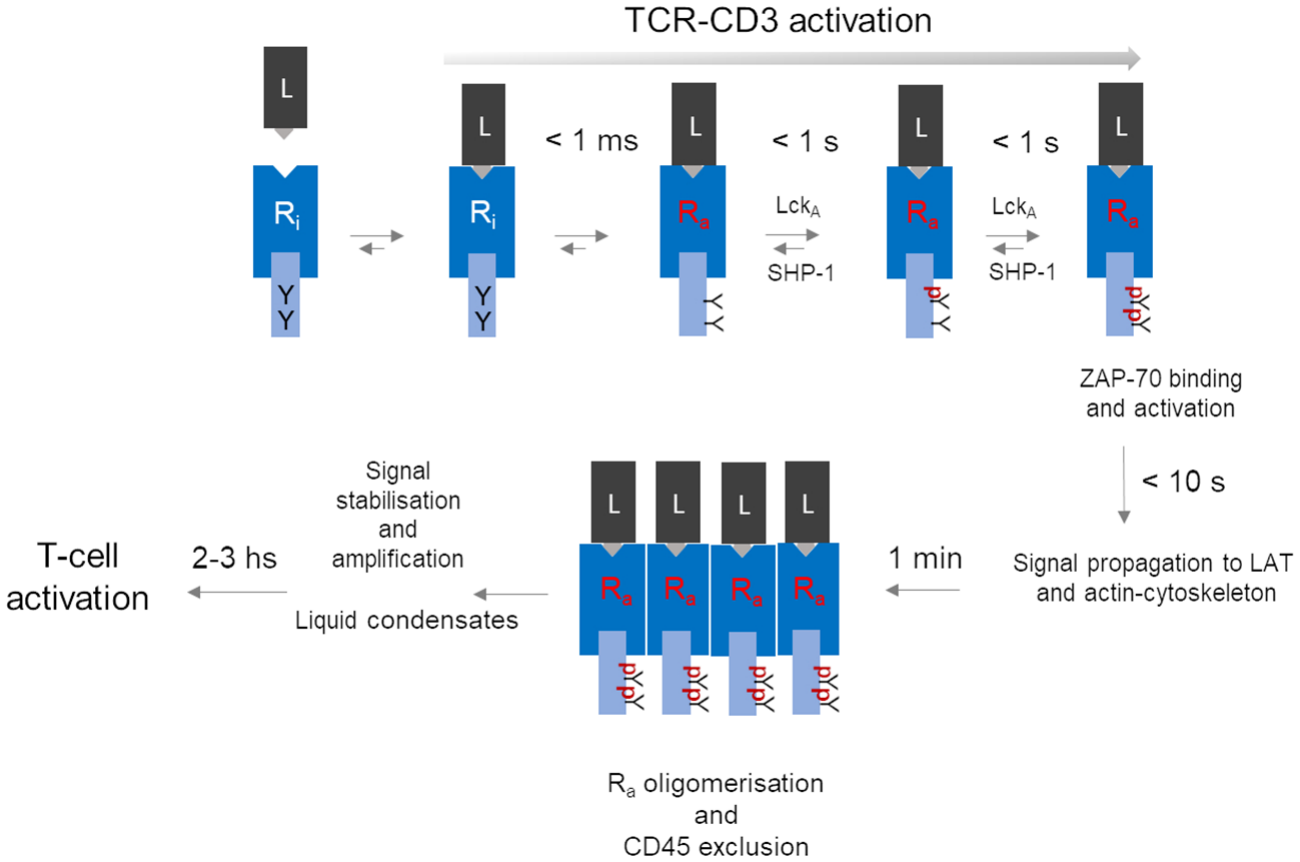
A hypothetical unifying model for TCR-CD3 activation leading to T-cell activation. The series of events depicted here is a summary of the process described in the paper, omitting for simplicity molecular details. The model contemplates a temporal cascade that initiates with an allosterically regulated-activation of the inactive TCR-CD3 (inactive Receptor (R_i_) induced simply by peptide-MHC (L) binding, leading to very fast (< 1 ms) tyrosine (Y) exposure in R_a_ to active-Lck (Lck_A_). Lck_A_ and SHP-1 negotiate ITAM phosphorylation (pY-ITAMs), eventually leading to 2pY-ITAMs if receptor occupancy is adequate and R_a_ can now bind and activate ZAP-70 to connect to the LAT protein scaffold for signal diversification (59). Data discussed in the text suggest that all sequelae of events take 10 s or so after ligand binding. The proposed model considers that only R_a_ can form tight clusters, while ZAP-70 (not shown) remains dynamically bound to R_a_. For simplicity, co-receptors have been omitted but if they are required for weak ligands, at a certain point they might depart from clustered R_as_. Other events should further stabilise the signal perhaps by condensation of signalling effectors near clustered R_as_. It is in the next few hours that key cell decisions will be made that involve nuclear events necessary for cell cycle entry and differentiation.

TCR-CD3 activation strictly refers to the molecular process by which p-MHC binding converts inactive TCR-CD3 into an active isoform able to deliver a signal to the cell interior (signal transduction). TCR-CD3 “signalling” is often used as a shortcut to mean not only TCR-CD3 “activation” but also signal amplification - *i.e.*, TCR-CD3 special property of multiplying the number of CD3 phosphorylated ITAMs -, signal stabilisation and intracellular propagation or even T-cell activation (**Figure 1**). This semantic lapsus overlooks the necessary spatiotemporal sequence of events (or cascade, **Figure 1**) and the specific physicochemical condition associated with membrane signalling, both being relevant for understanding the idiosyncratic molecular structure and functional behaviour of TCR-CD3. Thus, in a natural setting, where a p-MHC agonist is typically very scarce, TCR-CD3 activation and consequent signal firing likely is started by individual p-MHC-TCR-CD3 pairs (discussed below) at minuscule portions of the plasma membrane (< 60 nm^2^, as calculated from TCR-CD3 structure (11). However, signal firing by distant individual receptors may not add up effectively for achieving functionally relevant outcomes, due to the limited supply of enzymes and substrates and to stochastic noise (mechanical disturbance, protein crowdedness) that may inevitably slow down reaction rates. Clustering of individually activated receptors, a feature of virtually every membrane receptor (65, 66), provides great benefits to signalling: local increase of concentration of dedicated enzymes and substrates; the chance for weak non-Michaelis-Menten interactions favouring enzyme-substrate binding and rebinding; membrane lipid-dependent regulation; ligand rebinding (52) and protraction of allosterically-activated state of the receptor (65, 66). Early claims that TCR-CD3 forms abundant clusters at steady state (67) have been challenged by more perfected super-resolution imaging approaches (51, 68–70), including data analysis that limits particle over-counting (71). These investigations suggest that ligand-unbound TCR-CD3 is largely organised not into clusters (72). Thus, clustering could be a critical tipping point to increase and stabilise ITAMs phosphorylation rate and signal propagation (**Figure 1**).

TCR-CD3 emanates signals using molecular rules shared by other immune receptors (*e.g.*, BCR, FcRs, NKRs) but apparently not by classical membrane receptors activated by soluble ligands (*e.g.*, EGR receptor (EGFR), G protein-coupled receptor (GPCR), cytokine receptors). Thus, shortly after p-MHC binding, the tyrosine kinase Lck phosphorylates the ITAMs (each one containing two tyrosines (Y) in the cytoplasmic tails of the CD3 subunits ζ, ε, γ and δ (schematised in **Figure 1**). ITAM of different subunits are semi-conserved, with ζ harbouring three ITAMs and ε, γ and δ only one each, making a total of twenty tyrosines that could potentially be phosphorylated in a single TCR-CD3 molecule. ITAMs phosphorylated at a single tyrosine (pY-ITAM) cannot support productive cell activation because only phosphorylation of both tyrosines (2pY-ITAM) allows stable association and activation of ZAP-70 (73), a tyrosine kinase essential for TCR-CD3 signal propagation (73). ZAP-70 affinity for 2pY-ITAM is about 10 nM, much greater than the usual affinity range of p-MHC agonists suggesting signal stabilisation already at this early signalling stage. The degree of ITAM phosphorylation correlates with p-MHC binding duration (or dwell-time). ZAP-70 is mandatory for the positive selection of αβ T cells (74, 75), indicating that even weak p-MHC agonists induce sizable rates of 2pY-ITAM and of activated-ZAP-70 generation. Genetic data have shown that ITAMs multiplicity plays a quantitative role (76), strongly suggesting that the ITAMs are an expandable source of 2pY-ITAM and activated ZAP-70 generated at a rate dependent on ligand affinity and abundance. This rate is likely to be the most important parameter arbitrating whether a p-MHC agonist will make an αβ T cell either survive or clonally expand and differentiate or immolate. Estimates in individual cells and bulk populations suggest that it should take less than one second after TCRαβ binding to a medium/strong p-MHC agonist to induce pY-ITAMs elevation that shortly after activates major signalling pathways (*i.e.*, raise of IP_3_ and calcium) (59, 77) (**Figure 1**). Typical p-MHC agonists show dwell times of 5 to 60 seconds or longer, compatible with the timing of p-ITAMs detection. Weak ligands - *i.e.*, self-p-MHC inducing T-cell or thymocytes survival exhibit dwell-times as low as 100 ms or less (55). Thus, any molecular model of TCR-CD3 activation must be coherent with such a fast time scale. It has been suggested that very weak p-MHC agonists take longer times for pY-ITAM, leading to an approximate signal-storage phenomenon (78). However, the stochastic nature of signals just above the threshold may require time to attain sufficient synchrony of individual TCR-CD3 signal firing, eventually leading to pY-ITAM detection. Single-digit p-MHC agonists may suffice to elevate calcium concentration and activate Ras (79) and also induce the expression of genes activated by these pathways (80). However, priming a T cell for clonal expansion and full differentiation (or thymocyte death for negative selection) requires sustained engagement by p-MHC, forming persistent TCR-CD3 clustering and relatively stable signalling complexes beneath them (**Figure 1**) (59). Positive cooperativity between TCR-CD3 engaged with p-MHC monomers may be initiated by long-range effects, not necessarily mediated by lipid phase-like “rafts”, but rather by the TCR-CD3 “lipid fingerprint” (81, 82), followed by the multivalent assembly by lateral receptor clustering cemented more stably by direct protein-protein interactions and interactions with intracellular multi-protein signalling complexes (59). Such large molecular gathering forms 3D “signalling territories” at the cell-cell junctions or immunological synapses (IS) (83) between αβ T cells and APC (or target cells for cytotoxic αβ T cells). They are though to generate quasi-phases called “liquid condensates” (70, 84) ensuring sufficiently insulated environments for stabilisation and amplification of incoming signals.

### Coreceptors

Textbooks and review articles describing αβ T-cell activation often portray TCR-CD3 intracellular tails as rigid sticks freely floating in the cytosol, happily waiting to be phosphorylated by Lck brought by coreceptors CD8 or CD4 bound to MHC coincidentally with TCRαβ. Such representation implies that TCR-CD3 is a rigid protein complex barely capable, if not unable of MHC-restricted recognition and doing nothing to promote ITAMs phosphorylation, begging therefore a companion receptor to do it on its behalf. Such misrepresentation reduces to nil many valued publications of the past three decades that indicate a different setting, first and foremost that coreceptors play only a quantitative role in TCR-CD3 activation, hence they can be dispensable. Co-receptors come on stage to compensate for poor TCR-CD3 activation to achieve adequate αβ T-cell activation. Early genetic evidence in mice showed that CD8- or CD4-deficiency does not stop the development of mature MHC-I-restricted (cytotoxic) (85) and II-restricted (helper) αβ T cells (86), but reduces their number (85, 86), presumably compensating for co-receptor absence by selecting TCRαβ of higher affinity. Moreover, CD4-deficient mice restore normal numbers of MHC-II-restricted helper αβ T cells upon over-expression of a CD4 mutant unable to associate with Lck (87). Consistently, CD8 decreases the k_off_ of p-MHC binding to TCRαβ (88) and CD8 is fully dispensable for p-MHC agonists of K_D_ ≤ 5 µM (89). Feeble activation of CD8-deficient αβ T-cell by weak agonists can be largely compensated by increasing p-MHC concentration (*i.e.*, higher EC_50_ is observed) (89). These conclusions are backed by more recent data (90, 91). Coreceptor-bound Lck may act as an intracellular adaptor guiding preferential association to p-MHC-bound and activated TCR-CD3 (92, 93). This mechanism is supported by clever experiments showing that TCR binding to a weak p-MHC agonist experiences a sequential two-step increase in strength (94). The first binding component is sensed immediately after p-MHC ligation and it is coreceptor-independent, followed shortly after by a coreceptor-dependent binding increase. This second component disappears upon pharmacological inactivation of Lck (94), indicating that it is mediated by conformationally-open/active-Lck bound to the co-receptor. Moreover, super-resolution imaging suggests that co-receptor-unbound (or free) Lck augments in the proximity of TCR-CD3 soon after p-MHC binding, followed shortly after by the coreceptor-associated Lck (95). These data and the inherent bias for MHC-restriction of preselection TCRαβ repertoire indicate that CD4 and CD8 are a resource not for initiating but rather for invigorating, if needed, TCR-CD3 activation, incidentally making coreceptor-based TCR-CD3 activation models unlikely.

### Active-Lck, membrane-hung ITAMs, and ITAM phosphorylation

**Figure 2:**
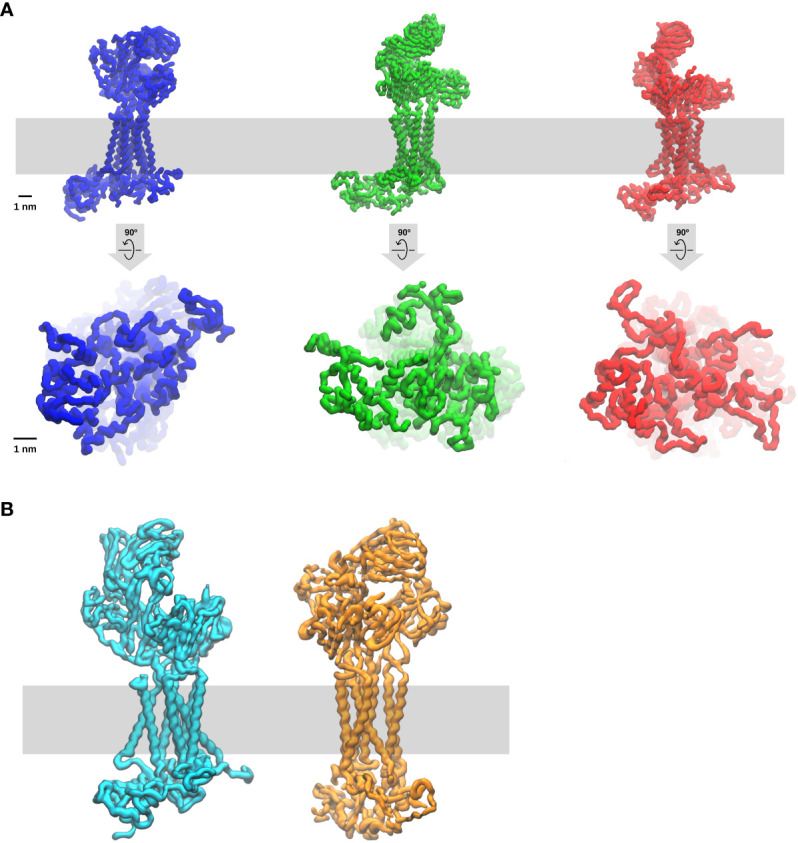
Molecular dynamic simulation of the entire TCR-CD3 complex (Courtesy of Dr Dheeraj Prakaash). The simulation of the full TCR-CD3 complex, including the CD3 intracellular tails, was for a total of five times for five µs and carried out according to the conditions described in (101). The lipid bilayer was composed of seven different lipids (including cholesterol and PIPs) and it is depicted as a grey band. Three snapshots are shown. **(A)** upper and lower panels are TCR-CD3 side and bottom (cytosol) views, respectively. Note the changes in the configurations of ectodomains, TMDs, and intracellular tails, indicate that TCR-CD3 is a relatively flexible complex with great potential for allosterically-regulated activation. Of interest is also the potential for correlated movements of these three TCR-CD3 domains perhaps exploited for the propagation of allosterically-driven changes in the three isoforms shown (“closed”, semi-open, fully-open). **(B)** TCR-CD3 side of two snapshots emphasising two extreme configurations one “open” (left) and the other “closed” (right). In the simulations, the transition from open to closed takes two-three hundred ns.

Akin to other Src-family kinases in other cell types, a fraction of constitutive enzymatically active-Lck is permanently present in T cells and thymocytes (96). The active-Lck pool is 40-60% of plasma membrane-resident-Lck (96, 97). Active-Lck is the net product of a highly dynamic equilibrium between Lck active and non-active (closed or auto-inhibited Lck) isoforms, the formation of the former being triggered and controlled by the membrane protein tyrosine phosphatase (PTP) CD45, which is also constitutively active (82, 96). Molecular details of this surprising mechanism have been recently documented (82). Such a condition was dubbed “stand-by” (96) to designate a state of cellular preparedness for TCR-CD3 activation, perhaps responsible for αβ T-cell sensitivity to low affinity ligands. It was suggested that ligand occupancy could initiate allosterically-regulated changes in TCRαβ that propagate to CD3 so that the ITAMs become accessible to active-Lck to initiate intracellular signal propagation (96). This idea prompts the question of whether ITAM tyrosines are somewhat concealed from constitutively active-Lck and become exposed only after TCRαβ engagement. An affirmative answer to this question is likely. More than twenty years ago, it was shown that the cytosolic tail of ζ (ζ_cyt_) behaves in solution like a random-coil (*i.e.*, devoid of secondary structure) that can be readily phosphorylated by Lck (98). ζ_cyt_ bound avidly to liposomes containing negatively-charged lipids, accompanied by bound-ζ_cyt_ showing some α-helix content and highly reduced phosphorylation by Lck (98). These observations are reminiscent of a paradigm-changing discovery in membrane biology made in the 1980s (99) revealing that some cytoplasmic membrane proteins contain unstructured clusters of basic and aromatic residues capable of mediating interaction with the inner leaflet of the lipid bilayer that are enriched with negatively-charged lipids (phosphatidyl-serine (PS) and phosphoinositide lipids (PIPs). Basic and aromatic residues strongly interact with PIPs (called also structural lipids) and with the bilayer hydrophobic core, respectively. Consistent with these notions, NMR studies showed that the CD3 ε-ITAM interacts with lipid micelles containing negatively-charged lipids, with the tyrosines partitioning dynamically into the lipid hydrophobic core (100), presumably reducing Lck access. FRET data in live cells showed that ε-ITAM interacts with the lipid bilayer inner leaflet of the plasma membrane (100). A cryo-EM structure of detergent-extracted TCR-CD3 complex is a major advancement towards understanding the mechanism of TCR-CD3 activation (11) that has revealed unsuspected features of the octameric complex. VαVβ is not standing vertically but leaning forward by an acute angle with respect to CαCβ. The CD3 dimers are asymmetrical arranged around CαCβ, with the upper loops of ε, γ and δ CD3 ectodomains making contacts with the distal loops of Cα and/or Cβ. The ζζ dimer is loosely bound to the rest of the complex via the transmembrane domain (TMD), making contacts primarily with αβ TMDs but surprisingly also with the TMDs of virtually all the other CD3 subunits (11, 101). These features indicate that TCRαβ and CD3 subunits form a highly interlaced quaternary structure with mutualistic contributions to TCR-CD3 topology that seamlessly connect αβ ligand binding site to the TMDs. This quaternary structure arrangement evokes opportunities for allosteric connections to promote ITAM exposure and phosphorylation upon ligand binding. However, the highly flexible CD3 tails cannot be seen in cryo-EM structures. To try and overcome this limitation, a computational tour-de-force by molecular dynamics simulations (MDS) of the entire cryo-EM structure with intracellular tails modelled as random-coils was carried out (101). This investigation has revealed that the cytosolic tails of all CD3 subunits interact with each other primarily by virtue of their random-coil nature, forming dynamic “skeins of tails” that are abutted against the plasma membrane (**Figure 2A**). CD3 ζ and ε (101) make the strongest contribution to membrane binding and show dynamic partitioning of some tyrosines in the hydrophobic core of the bilayer, in good agreement with the NMR data (100). The absence of PIPs in the modelled bilayer drastically reduces the formation of CD3 tail-skeins and interactions with the membrane (101). This study consolidated and extended observations suggesting that basic-rich stretches (BRS) preceding CD3 ζ and ε ITAMs interact with negatively charged PIPs (102, 103). Consistently, PIPs depletion in live T cells by the inositol polyphosphates Inp54p delivered to TCR-CD3 proximity leads to ITAMs phosphorylation by constitutive active-Lck without TCRαβ ligation (104), supporting the idea that ITAM unbinding from the membrane initiates TCR-CD3 signalling. The MDS study revealed also an unexpected movement of VαVβ bending down, with Vβ making dynamic contacts via charged residues with CD3γ, reaching a configuration of TCR-CD3 that makes it look “closed” (**Figure 2B**), suggesting perhaps a potential mode of ligand-induced allosteric activation. This MDS prediction is comforted by the latest available cryo-EM structure of TCR-CD3 embedded in a nanodisk that mimicks a membrane bilayer composed of a variety of lipids (105) and shows a “resting” configuration very similar to the “closed” configuration observed by Prakaash et al. (101). Also, the tail configuration may depend on changes in the relative positioning of the TMDs (**Figure 2A**) (101), in turn affecting the lipid composition in and around TCR-CD3 TMDs (or “lipid fingerprint” (81), including cholesterol (106) and ultimately PIPs (104), with the potential for altering CD3 tails interaction with the membrane and favour ITAM phosphorylation.

Ligand discrimination, a trait that αβ T cells are so gifted for, is key for preventing auto-immunity (107, 108) and effectively facing non-self (56). It requires a precise choice of a signal bandwidth, that should compromise between noise rejection (negative feedback) and reward (positive feedback) only for signals persistently levitating above the threshold (59). At steady-state, ITAMs partially accessible by Lck (98, 100, 101) experience low-grade/stochastic phosphorylation with pY-ITAM largely over-represented (**Figure 1**). The two net negative charges of pYs forbid interaction with the membrane hydrophobic core and may disturb BRS contacts as well, with some pY-ITAMs converted to 2Y-ITAMs that stably bind ZAP-70. If unopposed by a cytoplasmic PTP, such noise might become unstoppable. It has been suggested that the PTP SHP-1 controls ITAMs phosphorylation by Lck (109, 110) (**Figure 1**). Moreover, recent genetic evidence in mice agrees with this idea in that very weak agonists induce rapid SHP-1 association with pY-ζ-ITAMs and that mutation of all ζ tyrosines increases TCR-CD3 signalling and functional responses (111). This mechanism perhaps explains why pY-ITAMs are poorly, or not at all detectable at the steady state and how a signalling threshold is set by a dynamical antagonism between active-Lck and SHP-1 for ITAMs phosphorylation (discussed in Paster et al. (110), as part of the ligand discrimination mechanism (111). Presumably, 2pY-ITAMs increase above threshold drives ZAP-70 binding that decisively out-competes SHP-1 binding (a double-negative feedback) and protects 2pY-ITAMs from dephosphorylation, resulting in ZAP-70 enzymatic activation by Lck (a positive feedback) and rapid active-ZAP-70 accumulation (108). Such a mechanism could be the very first in a series of kinase-PTP control devises alongside the entire signal trajectory, initiating a proof-reading mechanism believed to implement αβ T-cell ligand discrimination (60).

## A critical appraisal of TCR-CD3 activation models

Structural and functional complexity is undoubtedly the main reason for contentious models on the TCR-CD3 activation mechanism. Some models privilege certain molecular or functional properties, yet neglect others, borrow in part paradigms of classical membrane receptors, or invoke entirely new paradigms. Unfortunately, little has been done to conceive discriminatory experiments and cooperation to nail down a unifying model. Currently, TCR-CD3 activation mechanisms (excluding coreceptor-dependent activation, as discussed above) can be segregated according to two major discordant structural notions: stiffness or flexibility, the former excluding allosterically-driven processes.

### Oligomerisation

TCR-CD3 oligomerisation (or clustering) causes a local increase of ITAMs concentration, raising by mass action the kinetics of their phosphorylation by Lck over the background (112). The simplest version suggested that TCR-CD3 activation is induced by binding to constitutively pre-clustered p-MHC (112). This and other oligomerisation models do not require TCR-CD3 structural flexibility (the receptor can be a “rigid body”). A major obstacle to activation by pre-clustered p-MHC is that αβ T cells comfortably respond to just a few p-MHC agonists dispersed among a huge excess of self p-MHC (79, 80, 113) and the probability of finding two or more rare p-MHC agonists associated at random in the same oligomer is vanishingly small. Also, crystal structures of p-MHC alone or complexed with TCRαβ are monomeric and accurate super-resolution imaging found no evidence for MHC-II clusters on agonist-loaded APC that otherwise stimulate T cells (114). Alternatively, p-MHC-TCR-CD3 pairs may laterally segregate and be drawn closer if receptor-ligand pairs of much longer size (*e.g.*, ICAM-LAF-1) form nearby (115). These effects can be driven by nanometre-scale membrane curvature resulting from tension for uneven membrane tethering (115). This model requires high agonist density, again antithetical to αβ T-cell high sensitivity and speed of TCR-CD3 activation (59, 77, 79, 80, 113). Moreover, genetic ablation of both major LFA-1 ligands, ICAM1 and 2 only attenuates TCR-CD3 signalling (116). The existence of steady-state pre-clustered TCR-CD3 is not supported by more recent super-resolution imaging investigations (51, 69, 70). Rather, single-molecule tracking coincident with early p-MHC binding suggests that monomerically-engaged TCR-CD3 can carry ZAP-70 (hence, it is already activated as a monomer) and experiences decreased lateral diffusion as compared to free-TCR-CD3, not due to clustering but presumably because already of this activated stage it is found bound to the actin cytoskeleton (51). Consistently, evidence suggests that clustering may be the consequence rather than the cause of TCR-CD3 activation (117). Moreover, soluble mono-dispersed p-MHC monomer alone suffices to induce early TCR-CD3 signalling, such as Erk activation (117). TCR-CD3 oligomerisation commonly observed at T cell-APC interfaces does not explain TCR-CD3 activation but is most likely a step following receptor activation (**Figure 2**) that is capital for triggering αβ T-cell activation.

### CD45 kinetics segregation (KS): can 6.6 nm stature difference decide whether TCR-CD3 is activated?

KS is an unconventional model based on mechanical force acting on a “rigid-body” membrane protein. KS is an elegant and intuitive model in cartoon representation. Moreover, its eccentricity in membrane receptor biology that addition does not require allosteric activation, proscribed by early crystallographic data (118), explains perhaps a relatively favourable reception by the immunologists’ community. However, at a closer look, the KS mechanism is non-trivial and rather convoluted. It requires some assumptions difficult to demonstrate, which together with existing data raise a number of disquieting questions. For KS to work, multiple TCR-CD3 bonding to p-MHC with adequate affinity must occur to enable the formation of relatively tight, nanometre-scale membrane junctions between T cell and APC membranes (119). KS implicitly assumes that TCR-CD3 and p-MHC are mono-dispersed at the steady-state but p-MHC-TCR-CD3 pairs rapidly oligomerise to achieve both tighter binding (by avidity) for relatively stable and laterally tight membrane tethering. Sufficient TCR-CD3 occupancy at small zipped-up areas should enable exclusion in a reasonable time of membrane proteins possessing long, encumbering, and rigid ectodomains (119, 120). The membrane PTP CD45 is a potential candidate for such lateral exclusion. KS relies on the basic notion that membrane receptor signalling requires a highly dynamical equilibrium of the contrasting action of protein tyrosine kinases (PTKs) and PTPs. Alteration of this equilibrium can tip the balance toward fast accumulation of ligand-bound receptor phosphorylation. While initial versions of KS suggested that CD45 controlled activation of Lck, a change of this paradigm (96, 121) made KS supporters propose instead that CD45 directly opposes ITAM phosphorylation by constitutively active-Lck (122). At steady-state, Lck-CD45 antagonism on Y-ITAMs would maintain pY-ITAMs ≅ zero. Reducing CD45 access to engaged-TCR-CD3 should lead to rapid pY-ITAMs increase, hence receptor activation. In simple terms, the higher and longer ligand receptor occupancy, the higher and longer CD45 is excluded, with consequent pY-ITAM increase. Earlier *in vitro* data suggested that CD45 dephosphorylates pY-ITAM (123). However, either CD45 genetic ablation or decreased gene dose or pharmacological inhibition of CD45 cannot be used to support KS. This is because, in agreement with the CD45 primary function discussed above (82), these manipulations strongly increase the pool of constitutively active-Lck (82, 121), hence TCR-independent pY-ITAM accumulation. Also, genetic evidence showed that CD45 ectodomain contributes to control constitutive levels of active-Lck (124), presumably by CD45 carbohydrates binding to membrane galectins (125, 126), a study that discouraged the use of chimeric CD45 with short ectodomains borrowed by other proteins to support KS. Evidence that the cytoplasmic PTP SHP-1 regulates pY-ITAM has also been gathered (109, 110) and strongly supported by recent data in mutant mice carrying CD3-ζ with all-tyrosine mutated to phenylalanine that show increased responses to weak p-MHC ligands (111). The authors show that SHP-1 is recruited pY-ζ soon after TCR-CD3 ligation, suggesting a direct control of pY-ITAMs by SHP-1, questioning the central assumption of the KS model. Super-resolution imaging has captured CD45 exclusion tens nm away from tight membrane junctions between the T-cell membrane and a surface densely coated with TCR-CD3 Abs, tens of seconds after cell spreading (127). However, this evidence is obtained under exceptional supra-physiological TCR-CD3 engagement in artificial conditions and similar evidence for CD45 exclusion is missing for physiological stimulatory conditions when the presentation of just a few agonists induces TCR-CD3 activation and very fast (77, 79, 80, 113). Idem for single p-MHC-TCR-CD3 pairs (51, 70). KS cannot easily explain how TCR-CD3 is activated by extremely weak ligands sufficient to guarantee thymic positive selection and mature αβ T-cell survival. The ectodomain of a major isoform of CD45 is ≈ 22 nm long (128), exceeding by 6.6 nm the sum of p-MHC and TCRαβ ectodomain. Since CD45 does not have a ligand on the APC, exclusion should be the result of mechanical compression exerted on CD45 ectodomain by the APC membrane bilayer (a relatively elastic surface). However, in the cartoon representation of KS the membrane and CD45 ectodomain are represented as rigid (*i.e*., surprisingly, the APC membrane is not at all deformed by CD45, so depicted as rigid, a rather unlikely condition). Thus, the 6.6 nm gap should determine the mechanical work required for CD45 ectodomain bending and exclusion, and the total energy of TCRαβ-pMHC bonding must exceed by a good margin the energy required for extruding CD45. According to KS, CD45 isoforms with ectodomains of considerably different length should differently affect T-cell activation. However, transgenic mice expressing only the longest or shortest CD45 isoform in comparable amounts show no functional effect on thymocytes development or activation of naïve and memory αβ-T cells (129). CD45 exclusion from c-SMACs can be observed hundreds of seconds after TCR-CD3 activation, generally in response to abundant agonist p-MHC amounts. However, cytotoxic αβ T-cells killing of target cells, which notoriously require very few agonist p-MHC, do not form c-SMACs and there are other instances in which αβ T-cell activation does not require c-SMACs. KS proponents have recently added a new twist to the model (128, 130), namely that ligand-engaged “small receptors” - *e.g.*, CD2 forms “close-contact zones” or a membrane zipper excluding CD45, a kind of signalling “heaven” where TCR-CD3 diffuses and gets activated. Besides the obvious inconvenience for TCR-CD3 competing for space in areas already densely occupied by CD2, such an idea is unsupported by evidence that thymocyte development and T-cell activation occur *in vivo* and *in vitro* in CD2 KO mice (131), corroborated by recent data in additional mouse mutants (132). Moreover, TCR-CD3 signals in artificial planar lipid membranes offer just cognate p-MHC. The KS (and oligomerisation) model does not explain how CD3 ITAMs detach from the plasma membrane to become accessible to active-Lck and how soluble p-MHC-tetramers or soluble mono-dispersed p-MHC induces ligand dose-dependent Ras activation (117). Most recent data suggests that CD45 exclusion serves the purpose of ligand discrimination (133), hence not TCR-CD3 activation *per se*. In conclusion, it stands to reason that KS is unlikely to explain TCR-CD3 activation.

### Mechano-transduction

It has been proposed that TCR-CD3 activation is triggered by forces pulling and/or pushing p-MHC-engaged TCR-CD3. The sources of force are T-cell motility and/or actin-myosin cytoskeleton dynamics acting directly on TCR-CD3 (134–139). The first proposition clashes in part with the notion that T-cell motility vis-à-vis the APC slows considerably and rapidly upon agonist-mediated TCR-CD3 activation (140), so force might not sustain signalling. Moreover, p-MHC presented on planar artificial bilayers can activate a T-cell that is kept essentially immobile for imaging purposes. With one notable exception (141, 142), crystal structures of many p-MHC-TCR αβ complexes do not show noticeable conformational changes occurring past the TCRαβ binding site (see below). However, the initial idealisation of TCR-CD3 quaternary structure has prompted some authors to propose that, if subject to force, TCR-CD3 could undergo conformation changes, otherwise invisible in crystal structures (135). Indeed, using experimental devices that generate ramping of traction force on p-MHC, TCR-CD3-dependent calcium rise is observed with force reaching tens of nanonewtons (138, 143). While attractive and intuitively simple, a serious caveat of mechano-transduction models is the lack of evidence that a uniform force (in time, space and ramp) of sufficient magnitude develops at the T-cell-APC contact sites after TCR-CD3 engagement. Only such ideal conditions should guarantee stereotypic conformational changes for TCR-CD3 activation. It is also unclear whether calcium rise depends on the ligation of single TCR-CD3 or multiples (already clustered) TCR-CD3, as in one experimental setting it was observed only after rapid serial TCR-CD3 pulling (143) and calcium rise was recorded considerably later after cell-cell contact (138). Recent data have suggested that the force developed at the T cell-APC interface upon TCR-CD3 ligation by p-MHC is considerably lower than the pulling force experienced by TCR-CD3 using artificial devices (144). Thus, although in a natural setting αβ T cell recognition of p-MHC undoubtedly occurs under some tensile force, its magnitude may not be as high as suggested (137). Actin-myosin cytoskeletal dynamics has been suggested to be the force provider (137). However, while pharmacological inhibition of actin-myosin dynamics in primary T cells does affect cytokine production, it does not affect very early TCR-CD3 signalling events such as ITAM phosphorylation (145) and actin appears to associate with TCR-CD3 already activated (51). Surprisingly, no test asking whether specific inhibition of Lck abolishes or decreases mechanical forces experienced by TCR-CD3 at the IS has been done. Mechano-transduction models cannot explain why soluble p-MHC small oligomers (tetramers, trimers, and dimers) or monomers (*i.e.*, conditions where no force is involved) activate TCR-CD3 (117, 146). Moreover, TCR-CD3 activation by just a few p-MHC agonists, as is often the case, may be perturbed rather than encouraged by force of certain intensity and reduce ligand discrimination (147). Force strength and direction of any origin (including long-range and slow lipid bilayer thermal fluctuation) at opposing cell membranes are likely to change randomly, and instead of inducing canonical conformational changes as suggested (135), it may rather perturb receptor-ligand engagement (147). However, under precise circumstances, the force could produce catch-bonding that reduces ligand off-rate, thus influencing ligand discrimination (138, 139), but catch-bonds do not occur with weak agonists (*e.i.*, self p-MHC) (55), making catch bonds not required for TCR-CD3 activation.

### Oligomerisation-induced allostery

Alarcon and co-workers provided the first experimental evidence that binding TCR-CD3 by soluble anti-CD3 Abs or p-MHC tetramers exposes in the CD3ε cytosolic tail a determinant situated in close proximity of the membrane (148). Such long-distance structural change upon receptor ligation is evidence for allosteric communication, prompting the authors to propose that TCR-CD3 signalling is allosterically regulated by conformational changes. The field was instantly divided into a few believers and many opponents and “wait-and-see”. Opponents thought that crystal structures are the ultimate revelation of protein mechanism of action (a pernicious misconception discussed in (149). Because conformational changes were not seen past the TCRαβ binding site in crystal structures, allosteric activation was unworkable. Certainly, opponents and sceptics were unaware that allostery must be first demonstrated empirically (by genetics and biochemistry approaches) and then studied by various means to understand which allosteric mechanism is at play (150). Indeed, NMR approaches can reveal distantly correlated dynamical changes that explain allosteric regulation without obvious structural changes, impossible to observe by conventional crystallography or cryo-EM. Advanced MDS approaches can also be useful to uncover distant correlated movements of the protein main chain induced by ligand binding to suggest the existence of allosteric trajectories (151, 152). Schamel and co-workers have suggested that ligand-induced TCR-CD3 oligomerisation with the precise lateral arrangement (“permissive geometry”) is promoted by pre-clustered p-MHC dimers on APCs and that this condition is responsible for inducing TCR-CD3 allosteric activation (153). In this model, p-MHC binding to TCRαβ alone does not trigger allosteric activation but ligand-induced TCR-CD3 oligomerisation does. “Permissive geometry” excludes therefore that monomeric p-MHC binding activates TCR-CD3. In essence, “permissive geometry” suggests that conformation changes are promoted by lateral interaction of the CD3 subunits triggered by pre-clustered agonists and propagate to the ε ζ intracellular tails. This idea is partly reminiscent of elegant models proposed by Bray and co-workers suggesting that oligomerisation increases ligand sensitivity by laterally spreading receptor activation (65). Although having some value for a more elaborated allosteric mechanism (see below), permissive geometry is crippled by the proposition that pre-clustered p-MHC agonists is mandatory for allosteric activation, a highly unlikely condition since the likelihood of finding at least p-MHC two agonist in the same hypothetical dimer of p-MHC should be extremely low, as discussed above.

### Evidence for allosteric sites in TCRαβ

Almost all crystal structures have shown that conformational changes are not found to significantly propagate beyond the binding interface (118). However, two NMR studies of mouse and human TCRαβ ectodomain unbound or bound to p-MHC independently showed compelling evidence for allosteric sites in Cβ (154, 155). Specifically, p-MHC binding produced dynamical changes in Vβ CDR3 residues that temporally correlated with dynamical changes in Cβ. Similar observations were made for MHC-I- and MHC-II-restricted TCRs and for different ligand affinities. These data constitute solid evidence for ligand-induced structural changes at long distances from the TCRαβ binding site β (155) without force or clustering. It is suspected that the relatively large interface connecting Vβ to Cβ (via the FG loop) contains the determinants that vehiculate such dynamic changes from Vβ to Cβ. Importantly, the Cβ residues that change dynamics upon ligand binding are exactly at sites that make contacts with residues of the CD3 ectodomains (155). Evidence for p-MHC-induced allosteric changes at Cα loops making contact with CD3 ectodomains has also been reported (141, 142). Comprehensively, these data suggest the tantalising hypothesis that the ectodomain of CD3 ε, γ and δ could be the intermediate receiver site sensing p-MHC binding to αβ for changes in the octamer TMDs and finally to the CD3 tails (see discussion in (117). Crystallography and other cryo-techniques that exploit ultra-low temperatures (- 190°C) can hardly capture ligand-induced dynamics gains characteristic of higher-energy (activated) states. They are therefore inadequate to reveal dynamically-driven (entropic) allostery, mediated by changes in protein flexibility as predicted theoretically in the 80s and now recognised to be much more diffused than originally thought, as demonstrated experimentally by NMR studies (156). It should therefore not come as a surprise that crystal structures of some liganded GPCRs that are certified allosteric receptors have at times failed to reveal expected structural changes, leaving room for entropic allostery. NMR can reveal allosteric connection by temporally correlating fast local conformational changes (at ps or ns timescales) occurring at sites tens of nm apart and inform on the remarkable speed at which allosteric changes travel along individual proteins or protein complexes – *e.g.*, one nm/µs (154, 157). Given the discrepancy between crystallographic data (and a recent cryo-EM structure (158) and NMR data, it is legitimate to suspect that TCR-CD3 activation relies on entropic allostery.

### Allosteric activation by monodispersed p-MHC monomers

Prompted by highly divergent models, a multi-pronged unbiased approach was set up using genetic perturbation of TCR-CD3 quaternary structure to probe for signalling alteration and integrated by biochemical approaches and MDS (117). The hope was to contribute to a unifying model. It was anticipated that small structural perturbations at particular sites, namely the TMDs of TCRαβ or CD3 subunits, could provide discrimination between “rigid-body” and allosteric models (for details of the rational, see (117). If allostery was found a plausible option, then other experimental criteria could be envisaged to include or exclude mechano-transduction and/or permissive geometry models. Surprisingly, mutations in TMDs of TCRβ or CD3ζ that minimally perturbed the stability of native TCR-CD3 quaternary structure, produced weak constitutive TCR-CD3 activation (*i.e.*, ζ phosphorylation without receptor stimulation) (117). These gain-of-function mutations did not promote receptor clustering *per se*, nor did they increase TCRαβ binding affinity or avidity for p-MHC. However, they did augment the proof-reading constant (k_p_), an indication of increased signalling efficiency (117), as if the mutations had pushed inactive TCR-CD3 (R_i_) towards an active (R_a_) isoform (**Figure 1**), somewhat reducing the activation energy required for this transition. Remarkably, mutation-induced basal activation of TCR-CD3, resulted in a perceptible increase in size distribution and frequency of TCR-CD3 clustering that was zeroed by pharmacological inhibition of Lck activity, suggesting that clustering was caused by TCR-CD3 activation and not vice versa (117). Importantly, all gain-of-function mutations reduced TCRαβ cohesion to ζ (and ε), as shown by a biochemical assay conceived to test changes (DDM-stability assay, DSA) (117). A similar behaviour of the gain-of-function mutations was observed for three TCRs of different specifcity. Such phenotype was corroborated by MDS studies of the mutants using the TCR-CD3 cryo-EM structure (117). This evidence made rigid-body models less likely. To exclude or include force and clustering, signalling recording (*e.g.*, Ras activation) and DSA were performed using p-MHC tetramers or rigorously controlled mono-dispersed p-MHC monomers and TCRs at the higher spectrum of affinity (*e.g*., K_D_ of 0.05 µM) (117). Both p-MHC monomers and tetramers induced Ras activation in a dose-response fashion in the absence of co-receptors, though tetramers elicited higher pErk amplitude and duration. Most importantly, both p-MHC tetramers and monomers loosened TCR-CD3 quaternary structure similar to the gain-of-function mutations, and such an effect was observed also if Lck was inhibited and at 4°C in intact cells or after TCR-CD3 solubilisation. Remarkably, activating anti-CD3 Ab binding to TCR-CD3 showed very similar allosteric changes (117). Ligand-dependent quaternary structure relaxation implies that p-MHC binding must necessarily affect contacts between TCRαβ and CD3 ectodomains, as suggested by the NMR studies (155), and ultimately perturb contacts in the TMD of the octamer complex that mediate ζζ (and possibly the other CD3 subunits) interactions with the other subunits (discussed in Lanz et al., 2021) (117). Comprehensively, the data gathered by this unbiased approach suggest that TCR-CD3 activation is controlled by an allosteric mechanism requiring only p-MHC monomer binding; thus independently of either force or oligomerisation or CD45 exclusion. Since pioneering studies rewarded with three Nobel prizes in 2013 (149), MDS has considerably advanced through vast improvements in software and access to powerful super-computers so that it is possible to obtain hundreds of ns-scale to single-digit µs-scale simulations in reasonable time frames to observe the dynamical behaviour of protein complexes embedded in a lipid bilayer, with constantly improving corroborative and precise predictive value (159). Recent MDS work using simulation times relatively long for all-atoms MDS (1 µs) has revealed p-MHC binding by different affinities to different TCRαβ consistently induce coordinated changes in dynamics in the main chain of Vβ and Cβ (152), in agreement with the NMR data. Similar effects were reproduced in the simulation of the entire TCR-CD3 ectodomain anchored to a lipid bilayer (151). Significantly, allosteric changes propagate at distances of several nm in just 1-2 ms (Kern and Zuiderweg, 2003; Natarajan et al., 2017), much faster than the shortest pMHC-TCRαβ 2D half-lives recorded thus far (*e.g.*, 50 – 100 ms). This key notion makes allosteric activation a valid mechanism to explain TCR-CD3 activation by very weak p-MHC ligands, and signal persistence by rebinding occurring faster than the disassembling of TCR signalling complexes (43). How ligand-induced changes at some contact sites of TCR-CD3 ectodomains (perhaps increasing CD3 conformational dynamics) lead to changes in TMDs to allow ITAMs phosphorylation remains to be deciphered. Slight modifications in TMD configuration may allow exchanges between lipids bound to TMD helices and bulk lipids, with potential for altering PIPs disposition and possible Y-ITAMs exposure. The signalling mechanism proposed for the EGFR also contemplates ligand-induced modifications in the configuration of the TMDs in the EGFR dimer (160). EGFR determinants in the cytosol side close to the plasma membrane carry stretches of basic resides that mutational analysis and MDS suggest to interact with PIPs (160, 161), a condition that may change upon ligand binding and cause a reorientation of the tween kinase domains (160). Consistent with the implication of membrane lipids in TCR-CD3 allosteric activation, mutations affecting cholesterol interaction with TCRβ TMD produce gain-of-function (106, 162). MDS indicate that changes in TMD inter-helical configuration may correlate with changes in the ectodomains and the CD3 tails (101) (**Figure 2A**), suggesting further mutational mapping strategies for augmenting or decreasing signalling. Models for allosterically regulated receptor tyrosine kinases (RTKs) and GPCRs able to bind different ligands of wide affinity differences, now integrate binding kinetics elements to better explain the ensuing biased agonism (163–165). The scenario suggested by Lanz et al., supported by NMR and MDS studies, implies that ligand efficacy for TCR-CD3 activation may be dependent on both allosteric changes and ligand affinity. There is indeed room for testing this idea using p-MHC that binds to TCRαβ with unorthodox orientations (166). In models of allosterically-regulated activation of TCR-CD3 by monomer p-MHC binding, ligand occupancy will determine the time of ITAMs accessibility to Lck and amplitude and duration of ITAMs phosphorylation. The “allosteric factor” could then be seen as an unsuspected manifestation of MHC restriction, in that some particular orientations of p-MHC over TCRαβ may elicit poor or invalid allosteric activation of the entire complex.

## Reconciling controversy?

Allosteric activation of TCR-CD3 dubbed some years ago as high unlikely (118, 167), has taken two-decades to mature into a plausible mechanism (101, 117, 148, 151, 152, 155, 162, 168). This is perhaps a sign that cartoon simplifications are often preferred to facts and interdisciplinary knowledge and that TCR-CD3 is a “smart receptor” (63), whose “reasoning” still holds secrets. The difficulty of easily accommodating oligomerisation, KS, and mechano-transduction as mechanisms that activate TCR-CD3 should be an occasion for conceiving a sensible unifying model, such as the one illustrated in **Figure 1**. This model orders in chronological order molecular events that begin with ligand-induced allosteric activation, the most hard-wired, fastest and finely tuneable mechanism for connecting the extra-cellular environment with the cell interior. Indeed, allosteric activation mediated just by ligand binding alone as the initiator of membrane receptor molecular activation has proved extremely valid in evolution as witnessed by thousands of different membrane receptors working according to its principles. Singly and sparsely firing signals by activated receptors (R_a_) cannot go very far in eliciting full cell responses and mandatorily require clustering, perhaps favoured by the actin cytoskeleton. R_a_ clustering is a sure fact in membrane biology and TCR-CD3 is no exception. Force cannot be completely excluded and together with CD45 segregation may create occasions for “positive effects” of biological impact, such as ligand discrimination. Considering the difficult conditions in which TCR-CD3 operates to activate a T cell, clustering must be of great value for extruding negative regulators and reducing physical and chemical noise from the membrane areas where and when action is going on for implementing signal propagation and diversification (59) and gene-wide activation required for αβ T-cell clonal expansion and differentiation. Such “signalling territories” is another welcome surprise for TCR-CD3 to face the best it can the uncertainty inherent with adaptive immunity.

## Author contributions

OA: Conceptualization, Funding acquisition, Investigation, Methodology, Supervision, Writing – original draft, Writing – review & editing.
